# Novel ‘mini-incision’ metacarpal fixation technique for long oblique and spiral shaft fractures

**DOI:** 10.1016/j.jpra.2024.11.007

**Published:** 2024-11-16

**Authors:** Safwat Ibrahim, Conor Cuggy, Jamal El-Deib

**Affiliations:** a Plastic & Reconstructive Surgery Department, Beaumont Hospital, Dublin, Ireland; b Royal College of Surgeons Ireland, Dublin, Ireland; c Plastic & Reconstructive Surgery Department, Prince Sultan Military Medical City, Riyadh, Saudi Arabia

**Keywords:** Hand surgery, Metacarpal fracture, Fracture fixation, ORIF, Cerclage wiring

## Abstract

**Background:**

Metacarpal shaft fractures account for 30 % of all hand fractures, and long oblique and spiral shaft fractures represent a significant quantity. Closed or open reduction and internal fixation is generally indicated for unstable fractures, rotational malalignment or significant metacarpal shortening. Various techniques can achieve appropriate fixation, though no single technique has been proven to be superior across all cases. The choice of fixation often depends on the fracture pattern, surgeon expertise and patient factors.

**Objectives:**

This large series aimed to show that our novel technique can achieve excellent clinical and aesthetic outcomes for metacarpal fractures despite being inferior in biomechanical studies.

**Methods:**

Briefly, 120 fractures in 115 hands were included in centres across Ireland and Saudi Arabia between 2016 and 2022. Inclusion criteria were patients with long oblique and spiral metacarpal mid-shaft fractures with significant displacement, metacarpal shortening and/or rotational malalignment. Fixation was achieved via cerclage wires inserted through a minimal dorsal incision. We examined the clinical and radiological outcomes of these patients.

**Results:**

We observed excellent results in our cohort, with 92.5 % of the patients obtaining full passive and active range of motion at the final follow-up. The mean qDASh score was 4.5. There was 1 case of malunion and 1 wire migration. No patients had cosmetic concerns regarding the dorsal hand scars.

**Conclusion:**

Our novel metacarpal cerclage can help patients regain excellent range of motion and avoid extended immobilisation. We believe that this method is technically simple, forgiving to mistakes, affordable and can provide excellent cosmesis

## Introduction

Metacarpal shaft fractures account for 6 % of all fractures in the human body and more specifically 30-40 % of all hand fractures.^1^, ^2^, ^3^ This places a significant burden on the society and healthcare resources. Metacarpal shaft fractures fall into the following categories: transverse, oblique, spiral and comminuted fractures. More specifically, the oblique and spiral fractures are subclassified into short and long fractures. Short fractures are less than double the width of the metacarpal shaft whereas long fractures are more than or equal to double the width of the shaft. The focus of this study is on the long spiral and oblique fractures.^1^

Several techniques have been described to address these metacarpal shaft fracture patterns. Fortunately, most of these can be treated conservatively.^1^^,^^4^ However, the indications for non-surgical and surgical management of metacarpal shaft fractures are still not clear in the literature. Publications on metacarpal shaft fractures have inconsistent indications on fracture fixation.^1^ Furthermore, the fracture must have no associated malrotation. This is consistently an indication for most surgeons and we agree entirely with this indication in our own practice.^1^ We assessed the rotation on a clinical basis alone. However, the acceptable fracture angulation and metacarpal shortening that will achieve adequate outcomes is debatable.^1^^,^^4^, ^5^, ^6^, ^7^, ^8^, ^9^, ^10^ We have described in detail the indications that the 2 main authors use in their practice (Table 1). Notably, the 2 main authors also included the absence of a sharp bone spike on the dorsum of the hand for conservative management.

**Table 1:** Non-operative criteria for metacarpal shaft fractures.

	Acceptable shaft angulation (degrees)	Acceptable shaft shortening (mm)	Clinical rotation	Dorsal bone spike
Index and Middle Finger	10-20	>/=4	None	None
Ring Finger	30	>/=4	None	None
Little Finger	40	>/=4	None	None

mm, millimetre.

We measured the angulation on lateral radiographs of the hand using mid-medullary measurement and measured the shortening by drawing a line through the 2 most distal points of the heads of the neighbouring 2 metacarpals.^11^

The most popular approaches for fixation use various K-wire techniques, lag screws, intramedullary compression screws or a combination of plates and screws.^1^, ^2^, ^3^^,^^11^, ^12^, ^13^, ^14^, ^15^, ^16^, ^17^ Furthermore, there are lesser documented techniques such as the use of circumosseous cerclage wire that have failed to gain traction despite the lack of high-quality evidence supporting any single fixation technique.^1^^,^^2^^,^^11^^,^^12^ Regardless of the approach used to repair metacarpal shaft fractures, it should be expected that most patients will achieve a full range of motion after rehabilitation if managed appropriately.^1^ If not treated appropriately, these fractures can limit the range of motion, lead to shortening, extensor lag and rotational deformities. The main disadvantages of using cerclage wires for metacarpal fractures include cerclage wire migration, due to the shape of the bone, and the perceived lack of rigidity in fixation.^11^^,^^12^

The main authors favour the use of circumosseous cerclage wire for metacarpal shaft fractures. This can be achieved successfully through a minimal 2-cm incision. This study analysed the results of a large cohort of long oblique and spiral metacarpal shaft fractures that were treated using circumosseous cerclage wire fixation across 2 sites. Transverse and short oblique and spiral fractures were not included as the bone contact surface is very small and deemed not appropriate for this fixation technique.

We believe that this novel technique demonstrates the excellent functional and aesthetic results that can be gained by using this circumosseous technique for this cohort of fractures.

## Patients and methods

All patients with oblique and spiral metacarpal fractures were treated with cerclage wire fixation by the 2 lead authors between January 2016 and December 2022. These treatments were carried out across 2 sites, St James Connolly Hospital, Dublin, Ireland and Prince Sultan Military Medical Hospital, Riyadh, Saudi Arabia. The records and clinical findings of these patients were reviewed retrospectively. The clinical and radiologic data were reviewed up to 6 months post-operatively. Patients with insufficient data and those lost to follow-up before 6 weeks were excluded from the series.

The following data were collected from the medical records, age, sex, comorbidities, site of fracture, number of cerclage wires places and any complications. Total active motion was used to assess the range of motion of the injured hand at the final follow-up (metacarpophalangeal, proximal interphalangeal and distal interphalangeal joints minus any extension lag). Additionally, we assessed the overall function using qDASH (quick Disabilities of arm, shoulder, hand) questionnaire. DASH question on the subject's opinion on appearance was used to assess the aesthetic outcome (1: normal, 2: virtually normal, 3: disfigured, 4: mildly unsightly and 5: very unsightly). All patients had X-rays taken at 6 weeks or their final follow-up appointment.

A total of 142 patients were included in the initial review of metacarpal shaft fractures. The final number of cases included those who were excluded following the exclusion criteria and 115 patients who were lost to follow-up, totalling 120 fractures.

### Patient selection and indications for surgery

All patients with long spiral and oblique fractures of a metacarpal shaft presenting to one of the lead hand surgeons met all the inclusion criteria. Our definition of shaft was taken from Orthopaedic Trauma Association as that part of the bone between the 2 end segments, with the end segment defined by ‘a square whose sides are the same length as the widest part of the epiphysis/metaphysis in question (Heim's system of squares)’.^18^ Long oblique and spiral fractures were defined as length of the fracture line being greater than twice the width of the metacarpal shaft. We measured the angulation on the lateral radiographs of the hand using mid-medullary measurement. We measured shortening by drawing a line through the 2 most distal points of the heads of the 2 neighbouring metacarpals and measuring the distance between the most distal point of the head of the fractured metacarpal and line drawn.^6^

Inclusion criteria included the following:

1. Presented within 4 weeks of injury.
2. Open or closed injury.
3. Adult patients (18 to 65 years of age).
4. Significant displacement and/or rotational deformity and /or significant angulation and /or bone spike dorsum hand.
5. Excluding the following
  a. Bone loss
  b. Intra-articular extension
  c. Lost to follow-up

The following protocol for the management of ‘long’ spiral and oblique metacarpal shaft fractures has been used by the 2 senior authors for the last 10 years. The authors do not specify the post-operative hand therapy regimes and the 2 units have used different protocols over the years.

### Surgical technique

**Figure 1:**
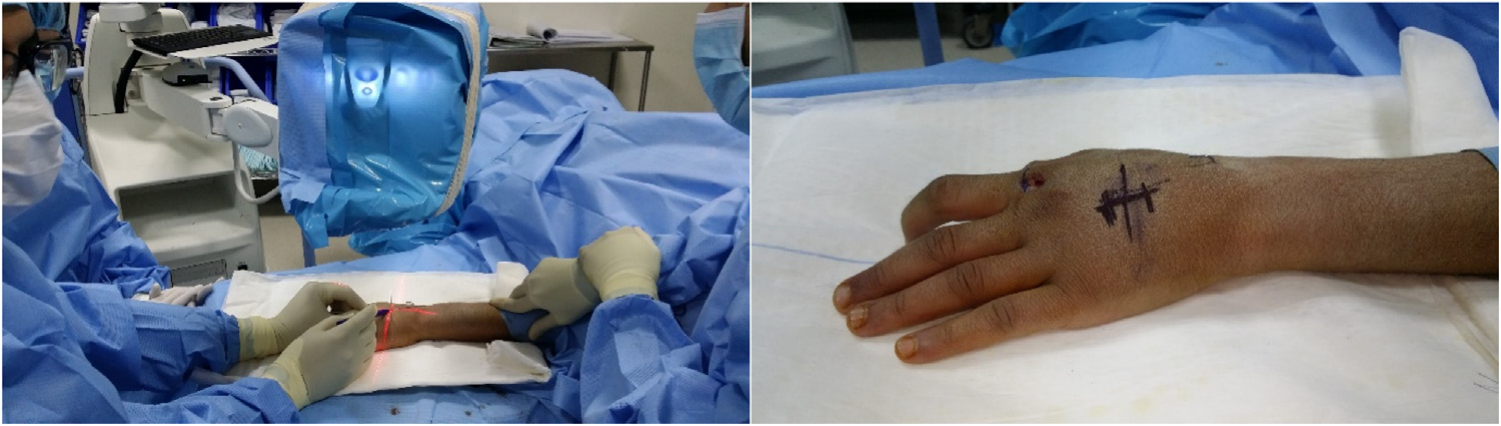
Marking the incision site.

**Figure 2:**
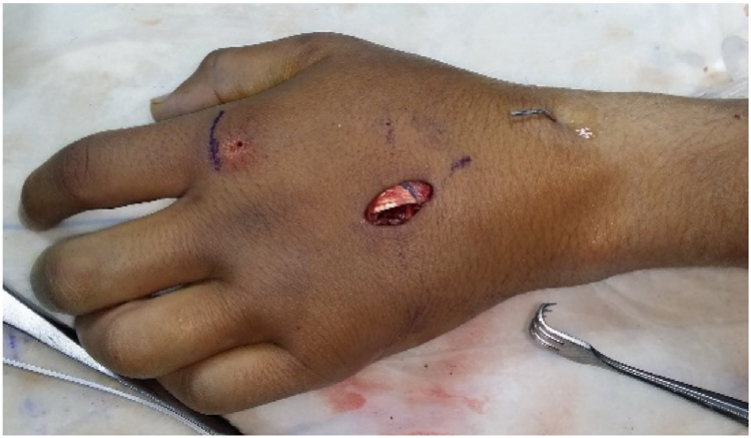
Mini-incision and dissection.

**Figure 3:**
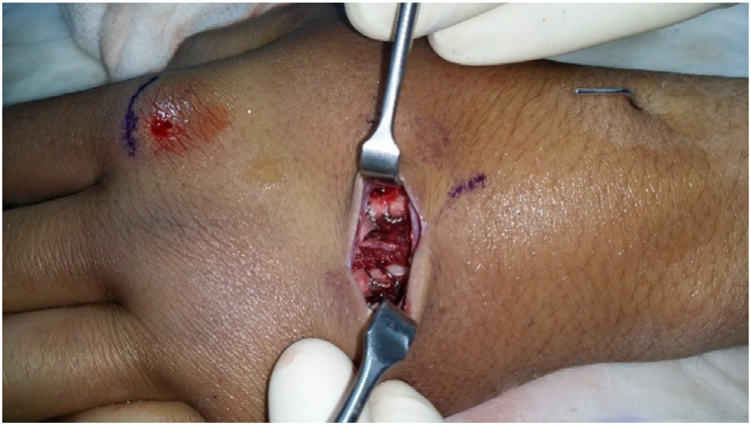
Cerclage wire fixation in the middle and ring metacarpal (intra-operative).

The lead authors performed all surgeries under either general anaesthesia or block. Fluoroscopy is initially used to mark the skin directly over the fracture site (Figure 1). Utilisation of 2 perpendicular K-wires and the fluoroscopy laser beam allows this to be carried out with precision (video 1). This allows a minimal 2-cm dorsal incision to be placed optimally (Figure 2). The fracture is then exposed via retraction of the extensor tendons using blunt catspaw retractors. The fracture site is thoroughly washed with normal saline. Any soft tissue including the periosteum is dissected free from the underlying metacarpal at the site of the fracture. Dental wire is passed around the metacarpal shaft. The 2 lead authors differ in their technique for this component of the operation. Both are demonstrated in the supplementary video. SI uses a pre-bent long metal needle in contrast to the aneurismal needle used by JE. The wire must be passed subperiosteally to avoid soft tissue entrapment inside the cerclage. Once the wire is around the bone, the fracture is reduced using traction. The wire is tightened onto the bone until anatomical reduction of the fracture is achieved (Figure 3). The reduction is then confirmed intra-operatively using fluoroscopy (Figure 3). A second and/or third cerclage wires are then placed around the metacarpal as required. Once the wires are tightened sufficiently, they are assessed for any rotational deformity and X-ray of the hand is taken again. If the reduction is satisfactory, the wires are cut and bent to the side of the bone to avoid injury to the tendon during movement. No bone scoring or drill holes are used with this technique. The dental wire used varied between 0.25 to 0.35 mm in diameter. If the fracture is inaccurately reduced, the wires can simply be untwisted one or 2 full rotations and re-tightened following adjustment to the reduction. For fractures of the distal and proximal shaft, a single K-wire is additionally placed transversely on the side towards the middle of the shaft (i.e. proximally in distal shaft fractures and distally in proximal shaft fractures) to prevent cerclage wire migration.

We close the skin in layers using absorbable 3-0 and 4-0 Monocryl. Post-operative splinting was carried out with a standard intrinsic plus dorsally positioned fibrecast splint. All fingers are initially immobilised. At day 3-5, all patients returned to the clinic for wound assessment, X-ray and hand therapy for fitting of a custom-made thermoplastic splint and early active mobilisation. Mobilisation progressed at hand therapy appointments on an individual basis.

## Results

A total number of 120 metacarpal shaft fractures in 115 patients were treated across the 2 sites. There was a total of 142 patients in the initial review of metacarpal shaft fractures, but 22 had insufficient documentation and follow-up. Ninety-six were men and 24 were women with a mean age of 33.5 (range 18-58) years. The fractured metacarpal was the index metacarpal in 11 patients, middle metacarpal in 37 patients, ring metacarpal in 59 patients and little finger metacarpal in 13 patients. The injuries occurred because of car accidents (n=44), industrial injuries (n=16), fighting (n=27) and falls (n=33). All patients had long, mid-shaft oblique/spiral metacarpal fractures. There were 2 cases of infection (both superficial) and 2 cases of chronic regional pain syndrome Type 1. There were no non-unions though there was 1 case of malunion. There was 1 case relating to the migration of the wire that required a correction surgery. There were no cases associated with impingement on the extensor tendons, extrusion or wire breakage. None of the other patients required removal of their metalwork.

**Figure 4:**
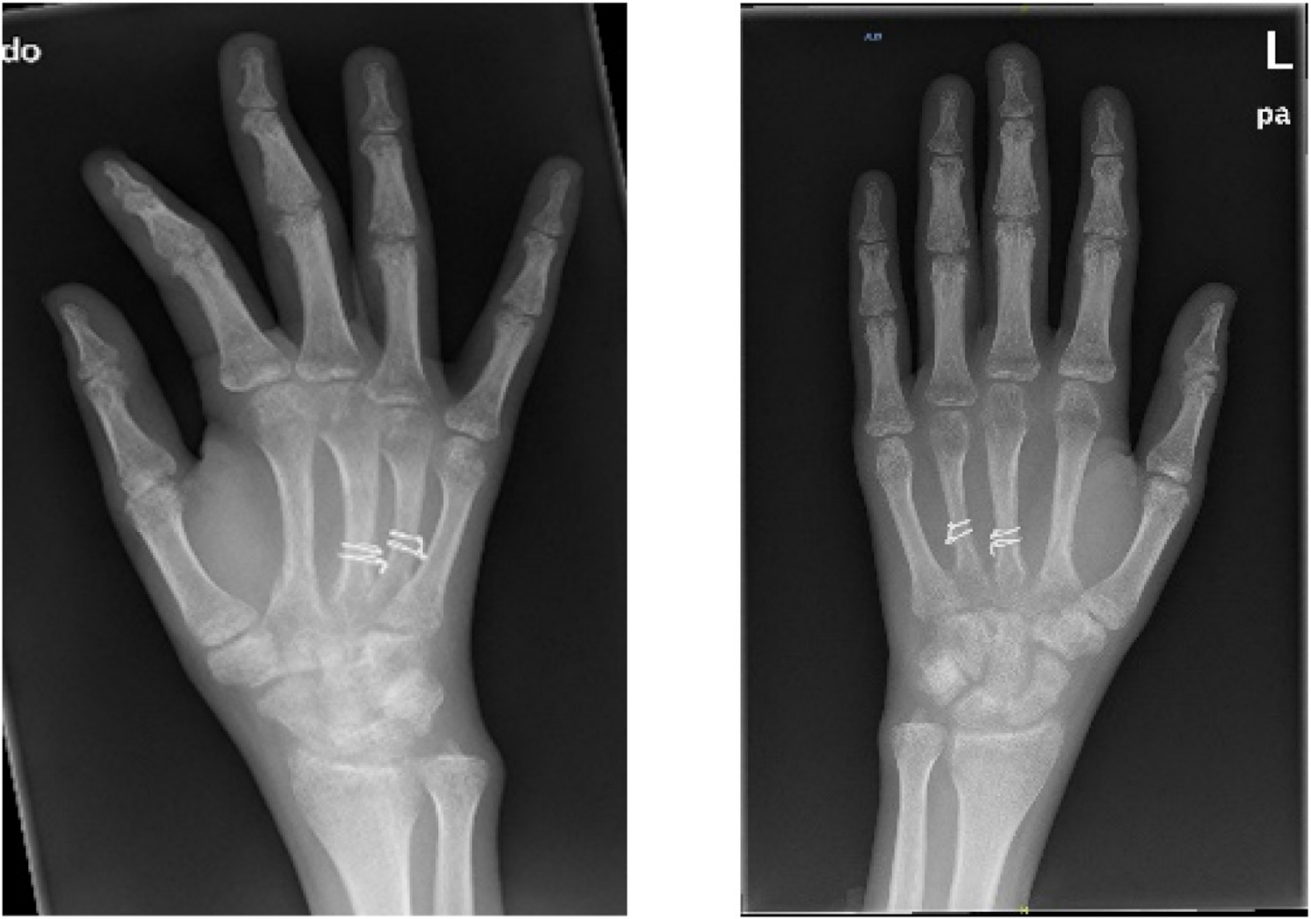
Post-operative X-ray of the middle and ring metacarpal shaft fracture at 4 months.

**Figure 5:**
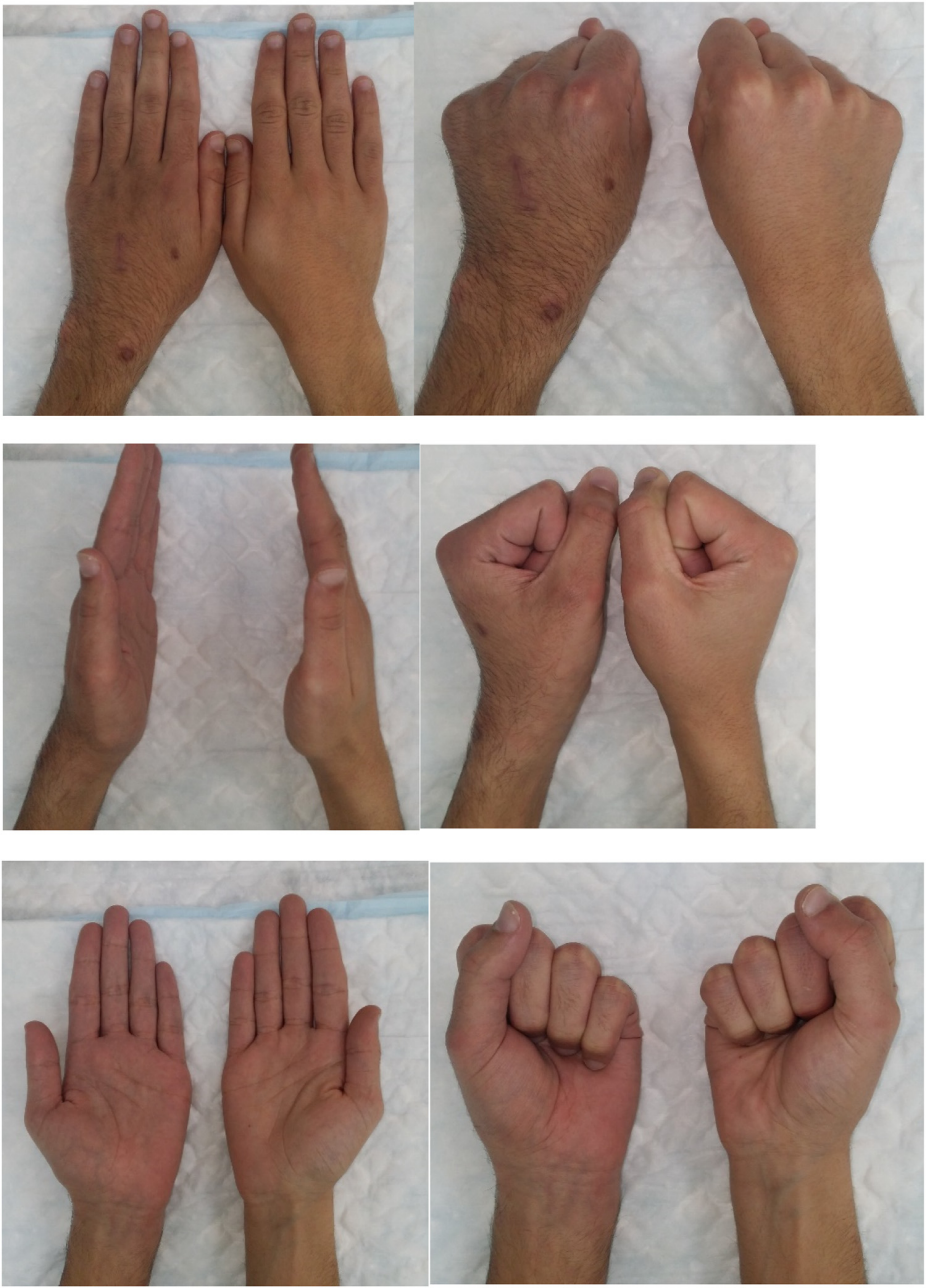
Post-operative images at the 4-month follow-up.

Overall, 111 (92.5 %) patients achieved excellent results based on the Strickland criteria by the final follow-up attendance with each patient achieving full passive and active range of motions with no deficit in tip to palmar crease (Figures 4, 5). The remaining 9 achieved good results based on the Strickland criteria. This was achieved following rehabilitation with hand therapy. The mean qDASH score was 4.5 (median 0) at the end of the follow-up. Approximately 33 % of the dorsal hand scars were slightly wide and hyperpigmented; however, only 4 (3.4 %) of the patients scored their aesthetic outcome as worse than ‘virtually normal’ (DASH score=2). The average time to return to work was 6 (range 2 to 10) weeks.

## Discussion

Among all metacarpal shaft fractures, long oblique and spiral shaft fractures represented a large proportion.^1^, ^2^, ^3^ We demonstrated our parameters for fixation of these fractures and used a novel technique for open reduction and internal fixation using cerclage wire with minimal access incision for several of these fractures and achieved excellent, consistent outcomes. This is an updated version of a technique that was originally described by Gropper and Brown in 1984 that has gained very little traction among the published works.^1^^,^^2^^,^^11^^,^^12^

In the literature, there are only 2 techniques using cerclage wire for fixation of metacarpal shaft fractures. In Gropper and Bowen's study, they described cerclage wire fixation in a cohort of 21 spiral and oblique fractures. They showed good outcomes; however, the main drawbacks included cerclage wire migration, due to the shape of the bone, and the perceived lack of rigidity in fixation. To address these issues, Gropper and Bowen used bone scoring prior to placement of the cerclage wire. Additionally, the cohort was immobilised for a minimum duration of 10 to 14 days.^12^ We believe that bone scoring to prevent migration is technically difficult and the risk of further cortical breaches on either side of the fracture site is high and unnecessary. The anatomical shape of the metacarpal should be considered with respect to wire placement. In contrast to the distal and proximal neck, which is thicker and varies along its length, the metacarpal remains thin and is relatively constant in diameter in the mid-shaft.

The other published work on cerclage wire fixation is from Al-Qattan in Saudi Arabia.^11^^,^^13^ In 2006, Al-Qattan used 2 to 3 cerclage wires for fixation but subsequently transitioned to using intraosseous loop wire in addition to their cerclage wires. In both the papers, the authors did not attempt to score the bone and mobilised all patients immediately without formal physiotherapy input to address the issues highlighted in Gropper and Bowen's study. Both papers showed excellent results without scoring the bone or immobilising his patients. All 19 of the included patients regained full range of motion and had no complications.^1^^,^^19^

We believe that early post-operative mobilisation is extremely important for rehabilitation of these fractures. Adequate stability is important to allow for this. The use of cerclage wire has a reputation of not being a rigid form of fixation. There is no doubt that cerclage wire is inferior in terms of bend, torsion and axial loading forces when compared to lag screws and plate and screw fixation devices in a non-clinical setting.^20^^,^^21^^,^^22^ However, a threshold for adequate stability is needed to allow for early mobilisation, and we have demonstrated clinically that cerclage wire fixation achieves this. Although there was one case of mild cerclage wire migration, it did not prevent the patient from achieving pain-free normal range of motion following rehabilitation. Thus, we do not believe that there is a need to immobilise these patients for extended periods.

Therefore, the choice of internal fixation should be based on the advantages and disadvantages of each technique. Evidently, lag screws are the strongest among all fixation techniques for oblique or spiral fractures of the mid-shaft; however, they can be technically demanding depending on the fracture pattern. Additionally, they are relatively expensive, require special equipment and are less ‘forgiving’ to mistakes. With lag screws there is only one optimal position, so mistakes mean that there are no chances of a second placement. The strength of K-wire fixation for long oblique and spiral fractures is insufficient to withstand the torsion load at the fracture site. The consequence of this can be rotational malunion of the fracture and eventually leading to a scissoring deformity. We do not consider K-wire fixation as the primary treatment for these fractures due to difficulty in achieving stabilisation of the rotational forces.^23^^,^^24^ Intramedullary screws are a newer method of fixation that is gaining traction for multiple different hand fractures. Although they can provide excellent results, the initial studies often quoted the issue of articular damage; however, this is not an issue in the metacarpal.^25^^,^^26^ However, we do not believe that intra-medullary screws or nails should be used for long oblique fractures. Additionally, they can damage the extensor tendon on entry which is more frequently an issue than we observed with our own technique.^25^^,^^26^

We believe the cerclage method for metacarpal shaft fractures is easy to perform and is very forgiving to mistakes. The wires can be easily adjusted and require a small incision. Plate fixation requires a longer incision and long plate to allow for bridging of the fracture site. This is associated with more extensive soft tissue dissection and postsurgical complications such as adhesions.^27^ Furthermore, no issues with adhesions were observed with use of the cerclage in our series.

In addition to the functional aspect, the hand is the most visible unclothed body part besides the face and thus patients seek as normal an appearance as possible. We believe that the impact of the aesthetic outcome should not be underestimated. The importance of this is exemplified by the increasing demand for hand rejuvenation procedures. We believe that our mini-incision allows for the best possible aesthetic outcome of all open reduction internal fixation techniques for metacarpal shaft fractures.^14^, ^15^, ^16^, ^17^

Limitations to this study include the fact that there are differing hand therapy protocols across both hospitals and across time within the study. Neither of the lead authors follow strict rehabilitation protocols if the patients mobilise early. This represents an uncontrolled factor in the outcomes for this cohort.

The fracture fixation differed depending on where the shaft of the fracture was located. There was no clear indication for when the addition of a transverse K-wire to prevent migration was warranted and was wholly dependent on the discretion of the operator of each case. Therefore, we did not segregate them into 2 separate groups. This may have affected the outcome of either group if we were to analyse them individually.

Additionally, the study provides no insight into the correct indications for surgery as we did not compare the cohort to any other method of conservative or surgical techniques. However, this study supports the non-inferiority of this method when performed adequately.

Lastly, this is a retrospective study. However, the data collected by both authors were succinct and abundant at the time of each patient's respective management allowing a thorough assessment of the dataset.

This novel mini-incision cerclage wire fixation technique for metacarpal shaft fractures has not been described previously. Overall, we demonstrated that it can provide excellent outcomes and is a very useful technique to have in one's surgical armamentarium. It is simple, more forgiving to mistakes, cheap and can provide excellent cosmesis from its minimal incision.

## Appendix Supplementary materials

Image, application 1
